# Cattle Bile *Arisaema* Aqueous Extracts Protect Against Febrile Seizures in Rats Through Regulating Neurotransmitters and Suppressing Neuroinflammation

**DOI:** 10.3389/fphar.2022.889055

**Published:** 2022-05-30

**Authors:** Fa-Zhi Su, Chen-Xi Bai, Yumeng Luo, Wen-Sen Zhang, Na Cui, Yang-Yang Wang, Yan-Ping Sun, Wen-Bo Zhu, Ming-Yang Zhao, Bing-You Yang, Hai-Xue Kuang, Qiu-Hong Wang

**Affiliations:** ^1^ Key Laboratory of Basic and Application Research of Beiyao (Heilongjiang University of Chinese Medicine), Ministry of Education, Harbin, China; ^2^ School of Traditional Chinese Medicine, Guangdong Pharmaceutical University, Guangzhou, China

**Keywords:** cattle bile *Arisaema*, febrile seizures, neuronal damage, neurotransmitter, neuroinflammation, anti-convulsant

## Abstract

Cattle bile *Arisaema* (CBA) is a traditional medicine used for the treatment of febrile seizures (FS) for thousands of years in China. However, its application is greatly limited due to cost reasons, and pig bile *Arisaema* (PBA) is the main commercial product instead. Additionally, the underlying mechanism of CBA for the treatment of FS still remains unknown. In this study, we investigated the anti-convulsant effect and potential mechanism of the CBA aqueous extract for the first time through a hot-water bath-induced FS rat model. Our results showed that pre-treatment with CBA dramatically lowered the incidence rate and generation times and prolonged the latency of FS. In addition, CBA effectively ameliorated neuronal damage and regulated neurotransmitter disorder induced by FS in the rat hippocampus. The enzyme-linked immunosorbent assay, western blotting, immunohistochemical, and qRT-PCR results exhibited that CBA suppressed the expression of GFAP, TLR4, NF-κB, HMGB1, NLRP3, TNF-α, IL-1β, and IL-6 and consequently inhibited the neuroinflammation induced by FS. Interestingly, although the CBA and PBA aqueous extracts possessed the same trend on the changes caused by FS, the improvement of FS by CBA is markedly better than that by PBA. These findings indicate that CBA exerts a protective effect on febrile seizures through regulating neurotransmitter disorder and suppressing neuroinflammation.

## Introduction

Febrile seizure (FS) is an epileptic convulsion defined by the International Alliance for the Prevention and Treatment of Epilepsy (Bozon et al., 2003). It is frequently encountered in infants aged from 6 months to 5 years with a prevalence rate of 2–5% (León-Navarro et al., 2015). It is often accompanied by fever and hippocampal injury, but there is no obvious evidence of central nervous system infection (Baram et al., 1997; Dong et al., 2011; Artis et al., 2012). Notably, most febrile convulsions are benign and self-healing, while recurrent FS or FS persistence not only contribute to long-term brain injury but also increase the risk of temporal lobe epilepsy (Gerges et al., 2003; Peng et al., 2004; Li and Xu, 2016).

For the past few years, researchers have explored the pathogenesis of FS and attributed the occurrence of FS to the disorder of neurotransmitters and neuroinflammation in the brain (Qu and Leung, 2008; Webster et al., 2017). Neuroinflammation is characterized by the increased release of pro-inflammatory cytokines or the decreased release of anti-inflammatory mediators in the nervous system (Dey et al., 2016). A lot of evidence demonstrated that increasing levels of pro-inflammatory factors in the brain result in FS generation, such as interleukin-1β (IL-1β), interleukin-6 (IL-6), tumor necrosis factor α (TNF-α), nucleotide-binding oligomerization domain (Nod-)-like receptor family pyrin domain containing 3 (NLRP3), and human high-mobility group box-1 (HMGB1) (Vezzani et al., 2004; Maroso et al., 2011; Mkhize et al., 2017; Webster et al., 2017; Zhong et al., 2018; Liu et al., 2020). Subsequently, these cytokines stimulate inflammatory signaling pathways including the toll-like receptor (TLR4) and nuclear transcription factor-kappa B (NF-κB) to produce an inflammatory response, and further cause fever, neuronal injury, and FS (Dubé et al., 2007). Furthermore, the disorder of neurotransmitters in the brain is also an important factor leading to FS, including excitatory neurotransmitters and inhibitory neurotransmitters. Increasing evidence clarify that a high expression level of glutamic acid (Glu) or a low level of γ-aminobutyric acid (GABA) can result in seizures (MacKenzie and Maguire, 2015; Freyd et al., 2017).

Clinical guidelines recommend the employment of antipyretic drugs combined with antiepileptic drugs to alleviate FS, such as diazepam, phenobarbital, and valproate (Lux, 2010; Huang et al., 2017). Unfortunately, the potential toxicity of these antiepileptic drugs to children is frequently greater than their therapeutic effects (Liu et al., 2020). They are not suitable for long-term use due to their serious side effects, for instance, inhibitory effects of diazepam on respiratory and electroencephalogram (EEG) activities (Mula, 2014; Blomberg et al., 2020), high mortality rate of valproate (Koziol et al., 2014), and cognitive impairment caused by phenobarbital (Ohlraun et al., 2013). Therefore, it is imperious to discover a drug with better effect and fewer side effects to fight FS.

More and more practice has proved that either single TCM, TCM prescriptions, or monomer compounds isolated from TCM exhibits a great therapeutic effect on FS. For example, Fangjing decoction, bear’s bile powder, cannabidiol in Cannabis sativa, and curcumin isolated from Curcuma longa can effectively prolong the latency, lessen the degree of FS, alleviate neuroinflammation, and balance the neurotransmitters in the brain to treat FS, respectively (Xu et al., 2018; Atabaki et al., 2020; Sun et al., 2020; Yu et al., 2020).

Bile *Arisaema* (BA) is a traditional common medicine to treat infant febrile convulsion clinically (Shan et al., 2021), it is the fermented product made of *Arisaema* rhizome and different biles including pig, cattle, and sheep, as documented in China Pharmacopoeia Committee. (2020). BA possesses bitter and cold properties, which can clear heat, dissipate phlegm, dispel wind, and relieve convulsions (Wu et al., 2020). Modern pharmacological studies have verified that BA exerts significant anti-inflammatory, antipyretic, analgesic, anti-convulsant, and sedative properties (Ahn and Je, 2011; Zhao et al., 2019). However, the effect of different fermented products on FS varies with the type of bile; studies have shown that cattle bile *Arisaema* (CBA) exhibited a better effect on FS than that of pig bile *Arisaema* (PBA) (Cui et al., 2021). The records of CBA can be traced back to the Song Dynasty, and ancient Chinese people usually adopted cattle bile as raw material for fermentation (Zhang, 1926; Xu, 1959; Li, 1977). But as time went by, the processing raw materials of BA were gradually replaced by pig bile. According to the market survey, pig bile is frequently served as the fermentation raw material of commercial BA due to the relatively cheap, large output, and easy access to pig bile (Tan et al., 2021; Wu et al., 2020). Additionally, no matter which bile is processed as raw material, the potential mechanism of BA in the treatment of FS is still unknown. Therefore, it is meaningful to identify a processed product of BA with the best effect on FS and explore the underlying mechanism.

In this study, we established an FS model in 21-day-old rats to explore the beneficial effect of commercial (PBA) and self-made BA (CBA) on FS and its underlying mechanism. In addition, the toxicology study of CBA and the qualitative analysis of bile acids in CBA were conducted to evaluate the safety of BA, and for offering guidance to clinical medication. This study provides the first evidence that CBA exerts a better neuroprotective effect against FS via suppressing neuroinflammation and regulating neurotransmitters than that of PBA. Our findings may provide reliable basis for further clinical applications of CBA in humans with FS.

## Materials and Methods

### Materials

Raw *Arisaema* samples (Batch No. 2005001) were supplied by Sichuan Neijiang Lianghui Pharmaceutical Co., Ltd., Longchang, Sichuan Province of China; they were authenticated as the dry tuber of *Arisaema* erubescens (Wall.) Schott by Professor Lianjie Su of Heilongjiang University of Chinese Medicine. Pig bile *Arisaema* samples (Batch No. 2011001) were also purchased from Sichuan Neijiang Lianghui Pharmaceutical Co., Ltd.; they were brown and black squares. The fresh bile of cattle (Batch No. 20201002) was collected from Yingtan Qiaokang traditional Chinese Medicine Co., Ltd., Yingtan, Jiangxi Province of China. The voucher specimens of the aforementioned three materials were deposited in the Key Laboratory of Basis and Application Research of North Medicine, Ministry of Education, Heilongjiang University of Chinese Medicine, Harbin, China.

### Chemicals and Reagents

Formic acid (LC-MS grade) was provided by Sigma (United States). Methanol and acetonitrile (UPLC grade) were bought from Thermo Fisher Scientific (Watham, MA, United States). Ultrapure water for all applications was prepared by Milli-Q water purification system (Millipore, Milford, MA, United States). All other chemical reagents were purchased from Tianjin Lihua Chemical Reagent (Tianjin, China) and they were all of analytical grade.

Reference standards containing cholic acid (CA), deoxycholic acid (DCA), glycochenodeoxycholic acid (GCDCA), glycodeoxycholic acid (GDCA), taurocholic acid (TCA), glycohyodeoxycholic acid (GHDCA), hyodeoxycholic acid (HDCA), chenodeoxycholic acid (CDCA), taurohyodeoxycholic acid (THDCA), taurochenodeoxycholic acid (TCDCA), and taurodeoxycholic acid (TDCA) were obtained from Shanghai Yuanye Biotechnology Co., Ltd. The purity of all the aforementioned reference standards was more than 98%, and they were used to qualitatively identify the bile acids in commercial and self-made Bile *Arisaema*, so as to compare the differences.

The ELISA kits of GABA (MM-0441R1), Glu (MM-0601R1), ALT (MM-21301R1), AST (MM-21304R1), BUN (MM-20555R1), NLRP3 (MM-1028R1), HMGB1 (MM-20429R1), TNF-α (MM-0180R1), IL-6 (MM-0190R1), and IL-1β (MM-0047R1) were provided by Jiangsu Meimian Industrial Co., Ltd. Yancheng, Jiangsu Province of China. The 10 and 200 μL automatic suction heads were purchased from NEST Biotechnology Co., Ltd. Wuxi, Jiangsu Province of China. Bicin, choninic acid (BCA, P00105), and radio-immunoprecipitation assay (RIPA) were supplemented by Beyotime (Shanghai, China). Primary antibodies containing rabbit anti-β-actin (AC026), rabbit polyclonal anti-NLRP3 (A14223), rabbit monoclonal anti-NF-κB (A18210), rabbit monoclonal anti-HMGB1 (A19529), rabbit polyclonal anti-TLR4 (A17436), rabbit polyclonal anti-IL-1β (A11370), and rabbit polyclonal-anti-TNF-α (A0277) were provided by ABclonal Technology Co., Ltd. (Wuhan, China). IRDye^®^ 800CW goat anti-rabbit IgG (H + L) secondary antibody was purchased from Beijing Bioss Biotechnology (Beijing, China).

### Drug Preparation

Preparation of cattle bile *Arisaema*: CBA samples were produced by traditional fermentation of the cattle bile with raw *Arisaema* by our laboratory according to the guidance of Chinese Pharmacopoeia and a traditional Chinese classical book (Xu, 1959; China Pharmacopoeia Committee, 2020). Mix fresh cattle bile with raw *Arisaema* powder in the ratio of 4:1 and stir until it becomes a paste. Then, pour the CBA paste back into the cattle gall and fasten the mouth to seal it. Hang it in a cool and ventilated place to avoid direct sunlight and temperatures higher than 20°C. After 100 days of fermentation, take out of the sample in the gall and crush it for use.

Water decoction preparation: In order to simulate the procedure of traditional Chinese medicine in treating patients with oral decoction clinically, the traditional decoction method was applied for the preparation of drugs. In brief, approximately 500 g of the pulverized CBA and PBA samples were respectively boiled with 5 L of water twice for 1 h (Cui et al., 2021). Cooled the extracts for several minutes and then filtered them through a muslin cloth. The filtrates were concentrated under low pressure at a final concentration of 1 g of crude drug per milliliter, respectively.

### UPLC-TOF-MS/MS Analysis of Bile Acids

In this work, the qualitative analysis of the bile acids was first comprehensively conducted using Ultra-high performance liquid chromatography coupled with time-of-flight mass/mass spectrometry (UPLC- TOF-MS/MS) due to the different processing excipients of the two drugs. About 2.0 g of CBA and PBA powders were weighed accurately into a 50 ml centrifuge tube and 20 ml 70% methanol was added, respectively, and the mixture was sonicated for 30 min. After the extracted solution was cooled to room temperature, we took an appropriate amount of supernatant into a 1.5 ml centrifuge tube, centrifuged at 13,000 r/min for 10 min, filtered the supernatant with a 0.22 μm filter membrane, and stored them at 4°C until sample injection. Appropriate amounts of the reference standards were separately weighed and dissolved in methanol to obtain the stock solutions, and they were mixed and diluted with methanol to prepare a final mixed standard solution. 100 μL of cattle bile sample and 900 μL of methanol were accurately sucked into a 1.5 ml centrifuge tube, vortexed for 2 min, then centrifuged at 13,000 r/min for 10 min, and filtered the supernatant with a 0.22 μm filter membrane for analysis.

The Waters ACQUITY UPLC system (Waters, MA, United States) coupled with AB SCIEX Triple TOF 5600 System (AB SCIEX, CA, United States) was adopted for data acquisition. A Waters ACQUITY UPLC HSS T3 column (1.8 μm, 2.1 × 100 mm) was employed to analyze at 35°C. Acetonitrile (Solvent A) and 0.1% formic acid in water (Solvent B) constituted the mobile phase for the analysis, and 0.3 ml/min was set as the flow rate. The injection volume of the sample was 5 μL, and the temperature of the autosampler was kept at 8 °C. A gradient elution method was performed as follows: 0–2 min, 5–35% A; 2–17 min, 35–75% A; 17–20 min, 75–95% A; 21–22 min, 5% A. In this study, both positive- and negative-ion modes equipped with an electrospray ionization (ESI) source were investigated for a more accurate identification of bile acid compounds. And the relevant mass spectrometer parameters in the experiment were as follows: scan range (m/z) from 50 to 1200 Da; ion spray voltage, 5 or–4.5 kV (for positive and negative mode, respectively); temperature, 550°C; curtain gas, 40 psi; ion source gas 1, 55 psi; ion source gas 2, 55psi. The Analyst^®^ TF 1.6 Software was used to operate the instrument and acquire all data.

### Animal

SPF-grade Sprague Dawley (SD) male and female rats at 21 days of age weighing 50 ± 10 g were used in the FS study. They were provided by Liaoning Changsheng Biotechnology Co., Ltd. (No. SCXK (LIAO) 2020-0001, Benxi, Liaoning Province of China). All the rats were kept in plastic cages under same standard laboratory conditions of temperature (23 ± 2 °C), humidity (50–60%), and an alternating 12 h light-dark cycle. The animals were subjected with food and water available *ad libitum*. All procedures were conducted according to Heilongjiang University of Chinese Medicine Laboratory Animal Manage and Use guidelines. The animal study was reviewed and approved by the Ethics Committee of Heilongjiang University of Chinese Medicine (approval number 2019121101) (Harbin, China).

### Toxicity Study

Raw *Arisaema* is a toxic medicine described as having strong irritant toxicity in Chinese Pharmacopoeia, but no matter, when processed by pig bile or cattle bile, the toxicity will be weakened (Su et al., 2022). In view of the model in this study and its clinical application, we chose male and female young rats as the subjects of the toxicity experiment in order to guide the clinical medication more accurately. On the basis of the guidelines of the chronic toxicity test (CDER, 1996; OECD Guideline, 2000; Louis and Hayes, 2001), we evaluated the toxic reactions caused by the dose of 10, 20, and 40 times of the clinical dose (7 g/kg, 14 g/kg, and 28 g/kg, respectively). In this part of the study, 40 rats (half male and half female) were divided into a control group, high-dose group (28 g/kg), middle-dose group (14 g/kg), and low-dose group (7 g/kg). The behavioral, neurological, and body weight changes of the rats after administration were monitored for 30 consecutive days, 24 h daily. Then, blood and internal organs were collected for biochemical and pathological analyses after the animals were anesthetized. The blood was centrifuged at 3, 000 rpm for 15 min to obtain the serum. All organs were wiped clean and weighed, determining the visceral index. The potential toxicity of CBA was evaluated by detecting serum aspartate aminotransferase (AST), alanine aminotransferase (ALT), blood urea nitrogen (BUN), and analyzing the pathological changes of organs.

### Establishment of FS and Treatment

Considering that febrile seizures are prone to occur in children between 6 months and 5 years old, we selected 3-week-old young rats as the research objective as previously recorded (Jiang et al., 1999). In our study, to mimic the clinical symptoms, the hot water bath immersion method was adopted to induce hyperthermic seizures on the basis of existing literature (Zhao et al., 2016). To determine the optimal temperature for the model, we tested several water temperatures to induce FS in the pre-experiment, 42.0, 43.0, 44.0, 45.0, and 46.0°C. Finally, 45°C was determined as the best modeling temperature, as it had the advantages of a high modeling success rate and low mortality compared with the other conditions. Exposure to hyperthermia was conducted by maintaining the water in a glass jar (35 × 35 × 50 cm) at a temperature of 45°C by placing it in a temperature-controlled water bath (Wu et al., 2017). The depth of the water in the glass jar was based on the fact that the rats could stand up against the side of the bath with only their head above water. The rats were immediately removed from the water after 5 min or once seizures occurred. The severity of the seizure behavior in rats was scored in accordance with the Racine scale with the following stages: 1) facial muscle twitch only; 2) nodding; 3) unilateral forelimb spasm; 4) bilateral forelimb spasm while standing; and 5) generalized tonic–clonic seizure (Racine, 1972). The convulsive behavior of the rats was monitored within continuous 3 h by at least two people. Furthermore, the latency to seizure, seizure duration, and core temperature were also measured in our study to evaluate the curative effect (Huang et al., 2015). After the observation, the rats were dried with a hair dryer until their fur appeared free of moisture, and then put back into their cages.

To explore the neuroprotective effect and underlying mechanisms of CBA and PBA in an FS-induced brain injury, a total of 70 rats at P21 were randomly divided into seven groups (*n* = 10 each group): vehicle group (control group); FS + vehicle group (model group); FS + sodium valproate group (20 mg/kg); FS + CBA groups, which were treated using high dose (2.8 g/kg), middle dose (1.4 g/kg) and low dose (0.7 g/kg) of CBA; and FS + high dose (2.8 g/kg) of PBA group. The rats were administered corresponding drugs by gavage 1 h before modeling, except the control group and model group. And an equal volume of normal saline was intragastrically administered into the rats in the control and FS groups. Then, rats in the control group were placed in the water bath at 37°C, while the other rats were put into water having a temperature of 45 °C until the seizures occurred. The hot water bath modeling was carried out once every 2 days, at a total of 10 times. Three hours after the last modeling, all rats were anesthetized with 25% ethyl carbamate and their unbroken brain tissues were collected for the subsequent experiments.

### Histopathological Study

In our study, brain tissue staining with hematoxylin and eosin (H&E) was employed to assess the neuronal damage. After the animals were anesthetized, the blood of all the animals was collected through the abdominal aorta for subsequent biochemical detection, and their complete brain tissues were removed. The brain tissues of the rats were fixed in 4% paraformaldehyde and embedded in paraffin. And then, coronal sections of 10 μm thick were cut using rotary microtome and stained with hematoxylin and eosin. The morphological changes of CA1 and CA3 neurons in the rats’ hippocampuses were identified as indexes to evaluate their pathological changes (Yu et al., 2020). The remaining brain tissues of the rats were snap-frozen in liquid nitrogen, and were preserved at −80°C.

### Immunohistochemical Staining

As mentioned previously, the hippocampus tissues of the rats were fixed in 4% paraformaldehyde, embedded in paraffin, and prepared as coronal sections. All of the sections were stained with brain-derived neurotrophic factor (BDNF) and glial fibrillary acidic protein (GFAP) antibodies to assess the activation of astrocytes and the expression of brain-derived neurotrophic factor before and after the treatment of FS (Sun et al., 2020). Section samples were imaged using a white light photo microscope (Nikon, Japan) and analyzed using Media Cybemetics software (United States).

### The Enzyme-Linked Immunosorbent Assay Analysis

In this part, the frozen hippocampal tissues were prepared into homogenates by a hand-held automatic homogenizer with ice-cold phosphate-buffered saline (PBS) containing protease inhibitor. The hippocampal homogenates and blood samples were centrifuged at 5,000 rpm or 3,000 rpm for 15 min at 4°C, respectively. The supernatant of the hippocampal homogenate and serum in blood were collected for the enzyme-linked immunosorbent assay. The levels of NLRP3, HMGB-1, TNF-α, IL-1β, and IL-6 in the hippocampus and serum were determined as per manufacturer’s instructions, respectively (Huang et al., 2015; Zhang, 2017; Liu et al., 2020).

### Measurement of Neurotransmitter Concentration

As described previously, the hippocampus of each rat was dismembered and homogenized, and after that, 2 neurotransmitters counting GABA and Glu were quantitatively analyzed (Sun et al., 2020). The concentration of these two neurotransmitters was determined through an ELISA kit according to the manufacturer’s instructions.

### Western Blotting Analysis

Refer to the previous literature reports (Vahdati et al., 2014; Zhang, 2017), the samples for the Western blot analysis were prepared as follows: The hippocampus was homogenized in a Radio-Immunoprecipitation Assay (RIPA) lysis buffer containing 1 mM phenylmethylsulfonyl fluoride (PMSF) on ice. The homogenates were centrifuged and the supernatants were collected to determine the protein concentrations. A BCA protein assay kit was used for measurement of hippocampal protein concentration following the manufacturer’s directions. The remaining samples were mixed with 5× loading buffer and then were incubated in boiling water for 10 min.

Equal amounts of proteins were loaded and separated using sodium dodecyl sulfate-polyacrylamide gel (SDS-PAGE) and then were transferred onto polyvinylidene difluoride (PVDF) membranes. After blocking, the membranes were incubated with the primary antibodies as follows: anti-β-actin (1:10000), anti-TLR4 (1:1000), anti-NF-κB (1:1000), anti-IL-1β (1:1000), anti-TNF-α (1:1000), anti-HMGB1 (1:1000), and anti-NLRP3 (1:1000) overnight. The PVDF membranes were washed 3 times using Tris-buffered saline Tween 20 (TBST) for 5 min and then incubated with anti-rabbit IgG-HRP conjugated secondary antibody (1:10,000) for 1 h at room temperature. The membranes were washed 3 times as described before. In the end, the protein bands were measured using Odyssey^®^ Clx imaging system and the data were analyzed via ImageJ software (Bethesda, MD, United States).

### Quantitative Real-Time Reverse Transcription PCR Analysis

The total RNA of the hippocampal samples was extracted using an RNA extracting solution under the guidance of the manufacturer’s instructions (Servicebio technology, Wuhan, China). RNA quality was measured using an ultramicro spectrophotometer (NanoDrop 2000, Thermo, United States). Then, RNA was reverse-transcribed into cDNA by Wuhan servicebio technology CO,. Ltd. (Wuhan, China). A quantitative-PCR analysis was performed *via* 2 × SYBR Green qPCR Master Mix (Servicebio technology, Wuhan, China) according to the following steps: 1) initial denaturation for 10 min at 95°C; 2) denaturation at 95°C for 15 s, anneal/extension at 60°C for 1 min for 40 cycles (Sun et al., 2020). The sequences of the primers for GFAP, BDNF, TLR4, NF-κB, TNF-α, and GAPDH are displayed in Table 1. GAPDH was selected as the internal control to homogenize the amount of cDNA in different samples. Finally, the relative mRNA expression levels were analyzed using the 2- △△Ct method.

**TABLE 1:** The sequences of primers for GFAP, BDNF, TLR4, NF-κB, TNF-α, and β-actin used in this study.

Gene name	Forward primers (5′-3′)	Reverse primers (5′-3′)
GFAP	AGA​GGA​AGG​TTG​AGT​CGC​TGG	GCC​ACA​TCC​ATC​TCC​ACG​T
BDNF	GTG​TGA​CAG​TAT​TAG​CGA​GTG​GG	ACG​ATT​GGG​TAG​TTC​GGC​ATT
TLR4	CCA​GGT​GTG​AAA​TTG​AGA​CAA​TTG	AAG​CTG​TCC​AAT​ATG​GAA​ACC​C
NF-κB	CAG​ATA​CCA​CTA​AGA​CGC​ACC​C	CTC​CAG​GTC​TCG​CTT​CTT​CAC​A
TNF-α	CCA​GGT​TCT​CTT​CAA​GGG​ACA​A	GGT​ATG​AAA​TGG​CAA​ATC​GGC​T
GAPDH	CTG​GAG​AAA​CCT​GCC​AAG​TAT​G	GGT​GGA​AGA​ATG​GGA​GTT​GCT

GFAP, glial fibrillary acidic protein; BDNF, brain-derived neurotrophic factor; TLR4, toll-like receptor 4; NF-κB, nuclear transcription factor-kappa B; TNF-α, tumor necrosis factor α; GAPDH, glyceraldehyde-3-phosphate dehydrogenase.

### Statistical Analysis

In our study, GraphPad 8.0 (GraphPad, La Jolla, CA, United States) was adopted for the statistical analysis. The continuous variables were presented as mean ± standard error of mean (SEM). The differences between the groups were performed through one-way analysis of variance (ANOVA). A p value less than 0.05 was considered statistically significant.

## Results and Discussion

### Identification of Self-Made CBA

Traditional Chinese medicine has a saying of “processed by cattle bile to remove dryness and maintain freshness” for bile *Arisaema* (Zhang, 1926). In order to explore which kind of BA has a better therapeutic effect on FS, CBA was made by fermentation of cattle bile and *Arisaema* in our laboratory. We determined the quality of CBA by identifying the properties according to Chinese Pharmacopoeia (2020 edition). CBA was a brownish-yellow powder, slightly fishy and bitter. Subsequently, a color reaction was carried out for chemical identification of the medicinal material. We took 2 ml of the filtrate of the product and after adding water, added 0.5 ml of newly prepared furfural solution and 2 ml of sulfuric acid. iFnally the junction of the two liquids exhibited a brownish red ring, which met the identification requirements of BA in Chinese Pharmacopoeia (2020 edition). Therefore, from this point of view, the fermentation method adopted in our laboratory is suitable for the preparation of bile *Arisaema* for follow-up research.

### Qualitative Analysis of Bile Acids Obtained From CBA

In this study, the bile acids in CBA and PBA were compared with standards using UPLC-TOF-MS/MS (Table 2). A total of eleven reference standards were analyzed in positive and negative modes. Interestingly, it was found that the separation effect and peak intensity in the negative ion mode were better than that of the positive ion mode. Furthermore, the optimal mobile phase, mass parameters, and gradient elution procedure were also investigated to obtained satisfactory chromatograms. The full scan mass spectrometry method was employed, and [M–H]^-^ was chosen as the precursor ions.

**TABLE 2:** The identified bile acids from CBA, PBA, and cattle bile.

No.	Compound	*t*_R_/min	[M-H]^-^(m/z)	CBA (*t*_R_)	Cattle bile (*t*_R_)	PBA (*t*_R_)
1	THDCA	4.03	498.2905	−	−	4.64
2	TCDCA	4.39	498.2892	5.84	5.81	5.85
3	TCA	4.46	514.2864	3.68	3.62	4.16
4	GHDCA	5.49	448.3063	−	−	5.47
5	TDCA	7.18	498.2909	7.34	7.48	6.77
6	CA	7.43	407.2797	7.58	−	7.37
7	GCDCA	7.68	448.6063	7.72	7.71	7.69
8	HDCA	7.90	391.2847	−	−	7.87
9	GDCA	8.22	448.3079	8.22	8.23	−
10	CDCA	10.68	391.2849	10.64	−	10.67
11	DCA	11.13	391.2852	11.23	11.12	−

(-) indicates that the component is not detected.

According to the current study, eleven cholic acids including free bile acids and conjugated bile acids were selected to compare the difference between CBA and PBA. The results showed that cholic acids in cattle bile existed mainly in combination with glycine and taurine before fermentation, while the type and content of free bile acids in CBA after fermentation with cattle bile increased significantly. This was consistent with the previous report on the changes of bile acids caused by pig bile fermentation (Li et al., 2018). By further comparing the chromatographic peaks with mixed reference standards of the samples we could find that TCA, TDCA, TCDCA, GDCA, and GCDCA existed in cattle bile, while after fermentation, free bile acids including CA, DCA, and CDCA arose in CBA. The difference was that, in the sample PBA, there were no corresponding chromatographic peaks at the retention time of CA and DCA, but the components of the unique series of hyocholic acids in pig bile containing HDCA, THDCA, and GHDCA were added. Furthermore, comparing the retention time of each component, we can find that the free bile acids possess a stronger retention performance than the conjugated bile acids, while the bile acid bound with glycine has a stronger retention performance than the taurine bile acids, and can achieve baseline separation. However, in consideration of acidity of taurine-conjugated bile acids, the chromatographic peaks of these components were widened greatly and showed a serious tailing phenomenon.

Although we accurately identified the changes of bile acids before and after fermentation, the change mechanism of bile acid components still remains unclear. It is worth noting that the content changes of these components in the bile of different species of animals on the market also needs to be systematically investigated in order to select the optimal processing raw materials. Additionally, whether the changes of the types and contents of different components in CBA and PBA will change the efficacy and action mechanism still needs more comprehensive experiments to verify and to screen better medicines for clinical application.

### Oral Toxicity Report of CBA in Rats

**FIGURE 1:**
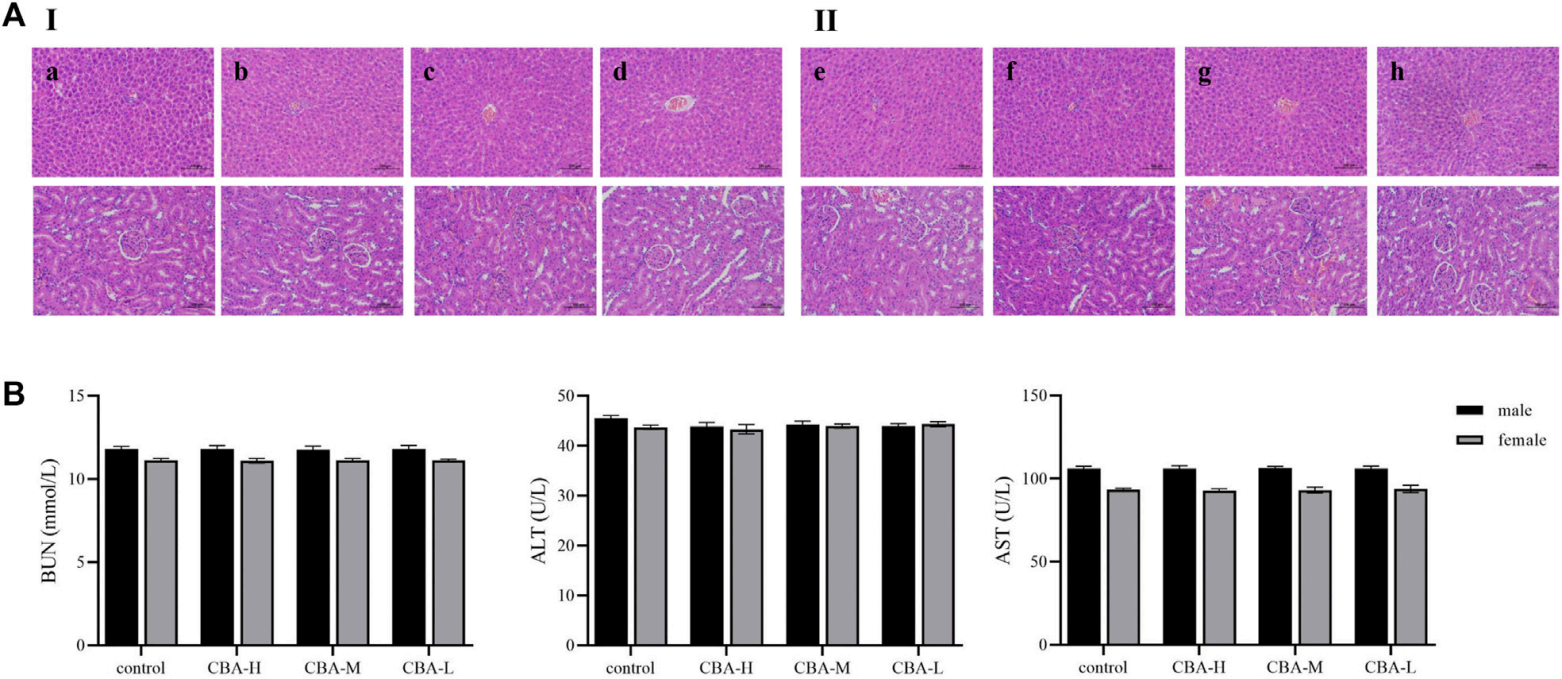
Toxicity evaluation of different concentrations of CBA after oral administration in rats. **(A)** The pathological changes of the liver and kidney in rats by HE staining: (I) male rats: (a) control, (b) CBA-H, (c) CBA-M, (d) CBA-L; (II) female rats: (e) control, (f) CBA-H, (g) CBA-M, (h) CBA-L. **(B)** Contents of BUN, ALT, and AST in rat serum.

According to the existing literature, oral administration of raw *Arisaema* can cause renal injury and different degrees of inactivity, bristling, shortness of breath, or dyspnea (Dong et al., 2015; Shan et al., 2021). However, the toxicity can be greatly weakened after fermentation with bile. In this study, we explored the oral toxicity of CBA, which was fermented from raw *Arisaema* and cattle bile in our laboratory. Due to the influence of administration volume and concentration, we did not measure the median lethal dose of CBA in the previous experiment. Therefore, we set up three different concentration administration groups (equivalent to 10, 20, and 40 times of the clinical dosage) to evaluate the possible toxic effects of oral CBA on rats, so as to ensure the safety of clinical medication. Following oral administration of CBA (7 g/kg, 14 g/kg, and 28 g/kg) for consecutive 30 days to male and female young rats, there was no phenomenon of death or significant toxicity indications in rats. Firstly, it was found that the physiological activities of rats were not affected before and after CBA administration compared with the control group. Furthermore, there was no significant difference in body weight and organ index among all the groups. Referring to serum biochemical parameters, we can find that there was no obvious difference in the serum levels of ALT, AST, and BUN between the CBA groups and the control group. On the basis of the results of HE staining of rat liver and kidney, no pathological changes were observed after an oral administration of different doses of CBA (Figure 1). All of the aforementioned results suggested that when the dose of CBA is 28 g/kg, it was still in the safe range and suitable for long-term use, which further explained that the toxicity of raw *Arisaema* was dramatically reduced after fermentation with cattle bile.

### CBA Effectively Inhibited the Onset of FS Induced by Hot-Water Bath

In the preliminary experiment, we determined 45°C as the best modeling temperature because of its low mortality and high incidence rate. Under such conditions, FS was successfully induced in the model group within 5 min, which was consistent with the previous literature which reported that hyperthermia induction can effectively promote seizures (Eun et al., 2015). There appeared various types of severe seizure-like symptoms, and progressed to tonic-clonic convulsions with a high core temperature. In addition, the rats in the model group were inactive and needed longer time to recover. In contrast, the rats treated with a high dose of BA (2.8 g/kg) had a shorter duration of convulsion, longer seizure latency, and a lower core temperature (Table 3, *p* < 0.01). Furthermore, the rate of the rats treated with a high dose of BA that exhibited generalized tonic–clonic seizures was lower than that of FS model rats. Interestingly, treatment with a high dose of CBA (2.8 g/kg) displayed a better effect than that of PBA (2.8 g/kg). Just as the data presented in Table 3, the differences between CBA and PBA were statistically significant (*p* < 0.05). Collectively, CBA treatment suppressed FS by reducing seizure severity, prolonging latency, and shortening the duration of FS, which was more effective than that of PBA.

**TABLE 3:** The effect of bile *Arisaema* (BA) administration on the latency, duration time, recovery time, and core temperature in a febrile seizure (FS) rat model.

Groups	Latency of FS (s)	Duration time (s)	Core temperature (°C)
Control	0	0	36.65 ± 0.4035
Model	206.3 ± 7.273^**^	480.3 ± 19.96^**^	41.55 ± 0.3719^**^
CBA-H	245.8 ± 8.804^###^	339.7 ± 10.42^###^	39.64 ± 0.8449^###^
CBA-M	228.8 ± 5.673	374.0 ± 10.91	40.88 ± 0.8311
CBA-L	210.1 ± 6.983	411.0 ± 14.71	41.09 ± 0.7340
PBA-H	230.0 ± 6.412^##▲^	377.9 ± 17.49^##▲^	40.43 ± 0.9673^##▲^
Sodium valproate	243.4 ± 10.381^###^	322.4 ± 12.94^###^	40.51 ± 0.6082^##^

The aforementioned data are expressed as Mean ± SD (*n* = 10). FS + CBA-H: 2.8 g/kg, FS + CBA-M: 1.4 g/kg, FS + CBA-L: 0.7 g/kg, FS + PBA-H: 2.8 g/kg, FS + sodium valproate: 20 mg/kg. ^**^ *p* < 0.01 compared to that of the control group; ^###^ *p* < 0.001 and ^##^ *p* < 0.01 compared to that of the model group; ^▲^ *p* < 0.05 compared to that of the CBA-H group.

### CBA Relieved the Histological Damage Induced by FS in Rat Hippocampus

**FIGURE 2:**
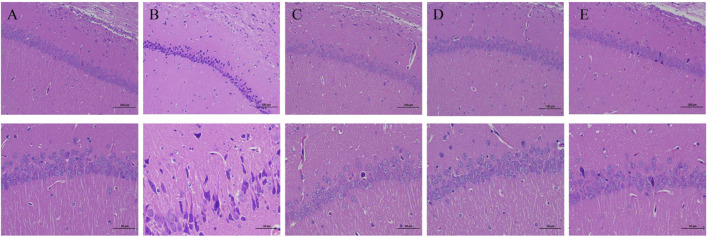
Cattle bile *Arisaema* (CBA) relieved the hippocampal neuronal injury by HE staining (200× and 400×). **(A)** control; **(B)** FS model; **(C)** FS + sodium valproate; **(D)** FS + CBA-H; **(E)** FS + PBA-H.

After hot water bath modeling, the amount and cellular morphology of the hippocampal neurons in the rats showed severe changes compared with those in the control group (Figure 2). In the hippocampal CA1 and CA3 areas of the rats in the model group, more neurons were shrunk, the cell staining was deepened, and the boundary between the nucleus and cytoplasm was unclear. The rats treated with a high dose of CBA (2.8 g/kg) displayed near total restoration, their hippocampal neurons were abundant and closely arranged, with a normal morphological structure, clear nuclear cytoplasmic boundary, and an obvious nucleolus. Nevertheless, although the dose of PBA was the same as that of CBA, PBA did not completely restore the changes of hippocampal neurons in the rats, and there were still a small amount of neuronal lesions. Accordingly, our experimental consequence showed that both CBA and PBA can reverse the damage of the hippocampal neurons caused by FS, but the same dose of CBA was better than PBA. These results further demonstrated that BA owned the anticonvulsant and neuroprotective effects in FS rats, but CBA worked better.

### CBA Regulated the Neurotransmitter Content in the FS Rat Brain

**FIGURE 3:**
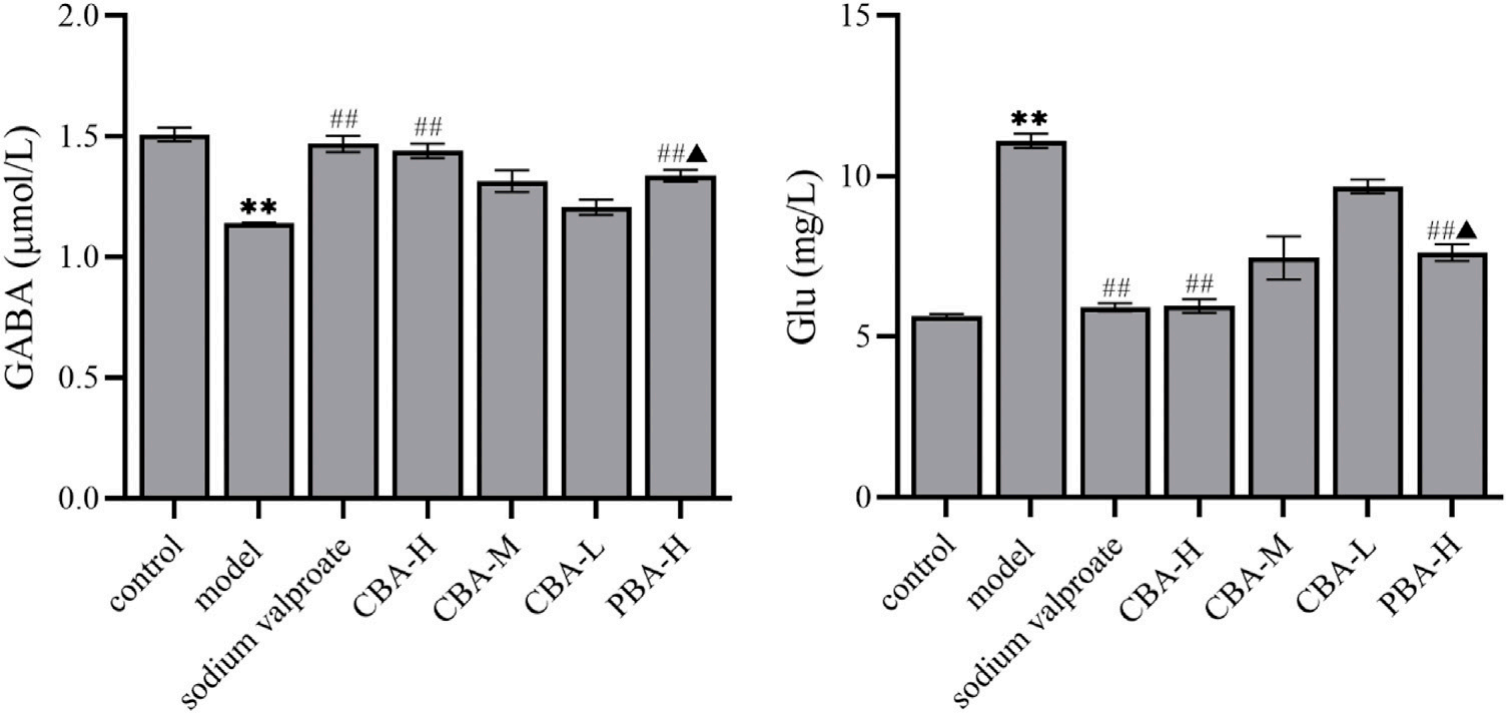
CBA regulated the neurotransmitter disorder in FS rat hippocampus. Data of neurotransmitter contents were calculated as the mean ± SEM. ^**^ *p* < 0.01 compared to that of the control group; ^##^ *p* < 0.01 compared to that of the model group; ^▲^ *p* < 0.05 compared to that of the CBA-H group.

An understanding of the neurotransmitter changes that are triggered by seizure activity is paramount for the identification of the mechanism to combat FS with treatment of BA. Looking back on previous studies, we can find that the seizure causes a reduction of GABAergic inhibition and an increase of glutamatergic excitation (Naylor et al., 2013; Glauser et al., 2016; Fernández-Sánchez et al., 2015; Lumley et al., 2021). Both the reduction of GABA and the increase of Glu are maladaptive and tilt the balance between excitation and inhibition toward more severe seizure activity. In this study, we measured the contents of two neurotransmitters (GABA and Glu) to explore whether the mechanism of BA in the treatment of FS is related to regulating the balance of excitatory and inhibitory neurotransmitters. The results showed that the onset of FS reduced the content of GABA and increased the content of Glu in rat hippocampus. In contrast, when treated with BA, the content of neurotransmitters in the rat hippocampus in the treatment group exhibited an opposite trend to that in the FS group (Figure 3, *p* < 0.01). It is worth noting that the ability of high-dose CBA to regulate neurotransmitter disorders in rat hippocampus is better than that of high-dose PBA (*p* < 0.05). To sum up, these data suggested that CBA may inhibit the development of FS by regulating the neurotransmitters in the hippocampus.

### CBA Inhibited Hippocampal Neuroinflammation in FS Rats Through Various Approaches

Numerous studies demonstrated that FS could induce an inflammatory response in the hippocampus, which released a great number of pro-inflammatory cytokines including TNF-α, IL-1β, and IL-6 (Maroso et al., 2010; Zhang et al., 2018). There is evidence that IL-1β secreted by the body can limit the ability of astrocytes to adsorb glutamate and down-regulate the content of GABA, resulting in the increase of brain excitability. The increase of its expression levels is related to the recurrence and duration of convulsion (Yu et al., 2012). IL-6 mostly comes from neurons, glial cells, microglial, and endothelial cells. It will lead to a large amount of IL-6 secretion when the body is damaged. Clinical studies showed that the level of IL-6 in the serum of children with FS was significantly higher than that of normal children, which confirmed that IL-6 was involved in the onset of FS. Additionally, when IL-6 was overexpressed in mice, it could enhance the convulsion susceptibility of mice by reducing the activity of the GABA receptor (Rose, 2012). It is reported that TNF-α can lead to FS in two ways. On the one hand, it can regulate the excitability of astrocytes, microglial cells and neurons, affect synaptic transmission, and lead to convulsion. On the other hand, it is an endogenous heat source with central thermogenesis, which can cause fever and induce FS (Aronica and Crino, 2011; Eun et al., 2015). HMGB1 protein is secreted by multifarious cells distributed in the brain, it can bind TLR4 to activate NF-κB pathway, and then promote the secretion of pro-inflammatory cytokines (Mazarati et al., 2011; Zhu et al., 2018). Beforehand, both animal models and clinical studies have confirmed that the HMGB1/TLR4 signaling pathway was involved in the onset of FS. The high expression of HMGB1 can significantly increase the frequency of seizures in children or rats (Sha et al., 2008; Maroso et al., 2010; Fu et al., 2017; Zhao et al., 2017; Walker et al., 2019). NLRP3 is an important composition of the NLRP3 inflammasome complex and is involved in neuroinflammation and epileptogenesis (Meng et al., 2014). It is a downstream signal of the NF-κB signaling pathway. When the body is stimulated, damage-associated molecules directly engage the TLR4 receptor and then quickly activate the NF-κB signal, resulting in the augment of the expression level of NLRP3 (Bauernfeind et al., 2009; Zhong et al., 2018). Recent experimental evidence verified that the NLRP3/IL-1β network contributed to the generation of FS, which provided a novel thought for the treatment of FS (Liu et al., 2020). According to recent research, CBA plays a better role in combating FS via inhibiting the inflammatory cytokines and excitatory neurotransmitters than that of PBA (cui et al., 2021). Herein, we comprehensively explored the mechanism of CBA in the treatment of FS by detecting the expression levels of inflammatory factors and related inflammatory signaling pathways.

**FIGURE 4:**
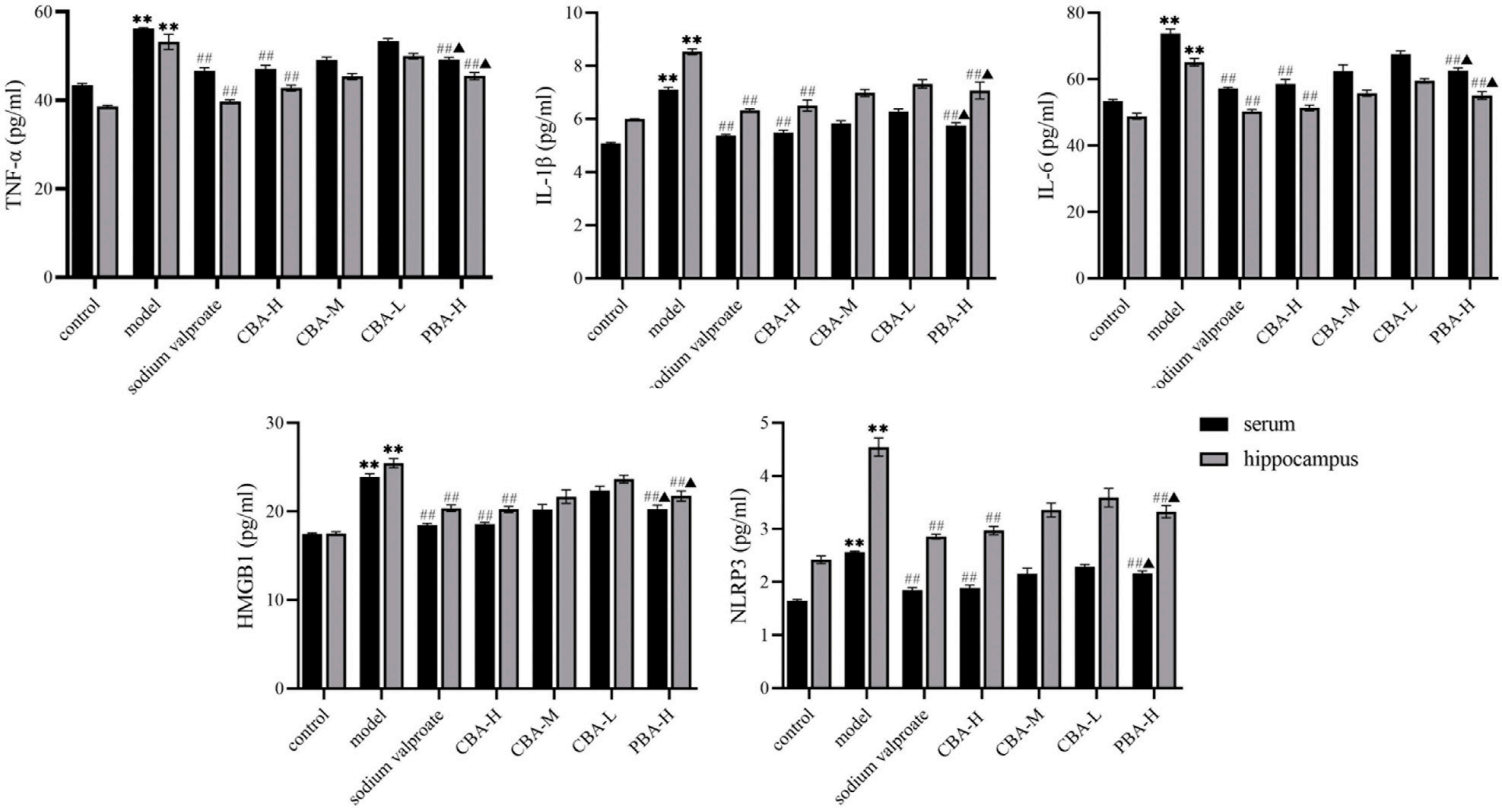
CBA inhibited the release of pro-inflammatory cytokines in FS rat serum and hippocampus. Data of pro-inflammatory cytokine contents were analyzed as the mean ± SEM. ^**^ *p* < 0.01 compared to that of the control group; ^##^ *p* < 0.01 compared to that of the model group; ^▲^ *p* < 0.05 compared to that of the CBA-H group.

Firstly, an ELISA assay was conducted to determine the TNF-α, IL-1β, IL-6, HMGB1, and NLRP3 expression levels in the hippocampus and serum. The data showed that the expression levels of TNF-α (Figure 4, *p* < 0.01), IL-1β (*p* < 0.01), IL-6 (*p* < 0.01), HMGB1 (*p* < 0.01), and NLRP3 (*p* < 0.01) were dramatically increased in FS rats compared to those in control rats. In contrast, after an oral BA pre-treatment, the high dose of BA significantly down-regulated the expression levels of the aforementioned inflammatory factors (*p* < 0.01). Interestingly, the ability of CBA to inhibit the expression of inflammatory factors was obviously stronger than that of PBA (*p* < 0.05), suggesting the better effect on FS of CBA. And it further suggested that CBA could play a crucial role in the treatment of FS by inhibiting the release of inflammatory factors.

**FIGURE 5:**
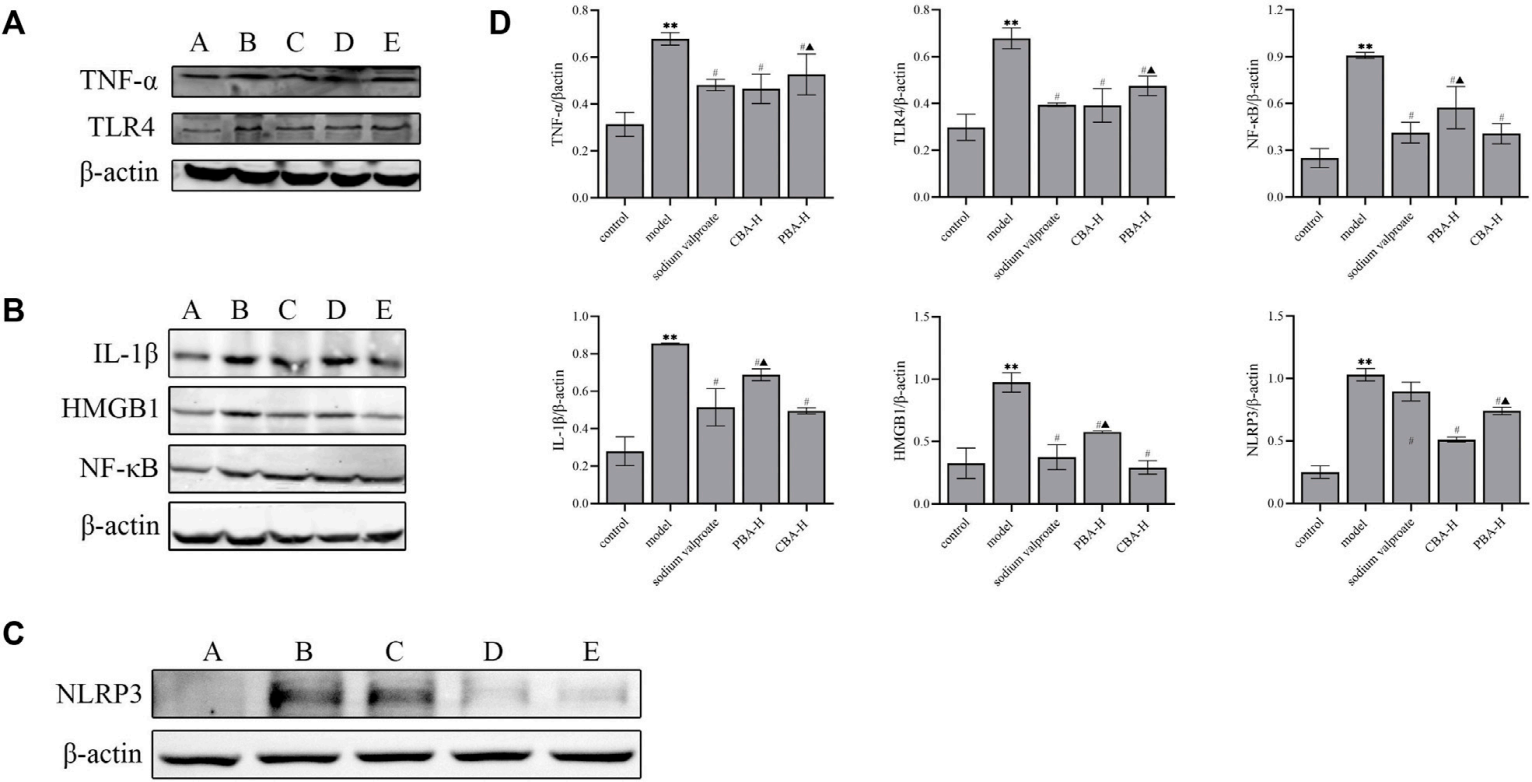
CBA exerted a neuroprotective effect on the FS model by inhibiting the activation of TLR4/NF-κB and NLRP3 inflammasome signaling pathway. **(A)** The expression levels of TNF-α and TLR4 proteins were analyzed by Western blotting: A) control; B) model; C) FS + sodium valproate; D) FS + CBA-H; E) FS + PBA-H. **(B)** The expression levels of IL-1β, HMGB1 and NF-κB proteins were analyzed by Western blotting: A) control; B) model; C) FS + sodium valproate; D) FS + PBA-H; E) FS + CBA-H. **(C)** The expression level of NLRP3 protein was analyzed by Western blotting: A) control; B) model; C) FS + sodium valproate; D) FS + CBA-H; E) FS + PBA-H. **(D)** The semi-quantitative analysis for Western blotting. Data of relative protein expressions were exhibited as the mean ± SEM. **p < 0.01 compared to that of the control group; ^#^ *p* < 0.05 compared to that of the model group; ^▲^ *p* < 0.05 compared to that of the CBA-H group.

Secondly, to demonstrate the therapeutic effect of CBA at the protein level, the Western blotting analysis was conducted on rat hippocampus from each group (Figure 5). The original western blotting data are available in the Supplementary Material. It was found that the protein expression levels of TNF-α, IL-1β, HMGB1, NLRP3, TLR4, and NF-κB were markedly increased in the hippocampus of FS rats (*p* < 0.01) compared to those of the control group, while greatly reduced in BA treatment groups (*p* < 0.01). Interestingly, the trend of the high-dose CBA down-regulating protein expression was more obvious than that of PBA (*p* < 0.05). These results revealed that CBA could prevent FS by inhibiting TLR4/NF-κB and NLRP3 inflammasome signaling pathway.

**FIGURE 6:**
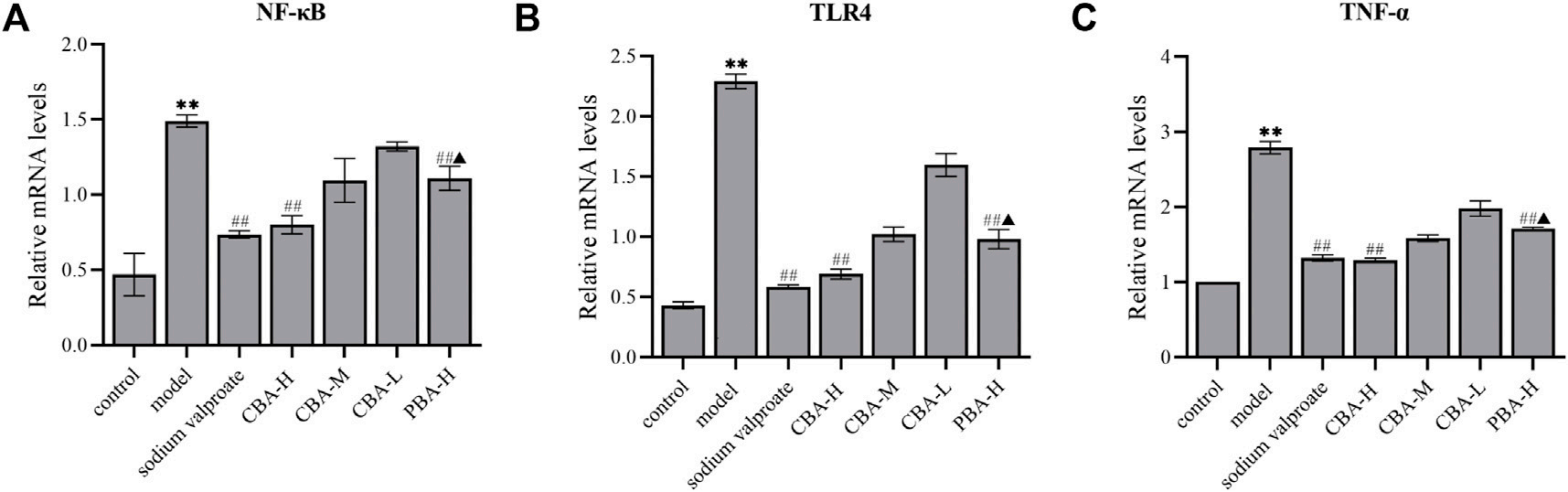
CBA suppressed the mRNA expression of NF-κB **(A)**, TLR4 **(B)**, and TNF-α **(C)** in the hippocampus of FS rat model. **(A)** The mRNA expression of NF-κB in the hippocampus; **(B)** The mRNA expression of TLR4 in the hippocampus; **(C)** The mRNA expression of TNF-α in the hippocampus.

Finally, we verified the curative effect on FS of CBA by inhibiting the TLR4/NF-κB signaling pathway *via* qRT-PCR. The experimental data remaindered that FS resulted in the dramatical increasing of the mRNA expression of TLR4, NF-κB, and TNF-α in the hippocampus (Figure 6, *p* < 0.01). As expected, a high dose of CBA effectively prevented the increased expression of genes related to the inflammatory signaling pathway (*p* < 0.01), which was better than that of PBA (*p* < 0.05).

In conclusion, we performed various approaches to verify the inhibition of hippocampal neuroinflammation of CBA for the first time. As documented in previous studies, FS induced an inflammatory response in the hippocampus and promoted to release a large amount of pro-inflammatory cytokines (Cartmell et al., 1999). These cytokines play an important role in neuroinflammation and seizure onset. In our study, the expression levels of TNF-α, IL-1β, IL-6, HMGB1, NLRP3, TLR4, and NF-κB were dramatically reduced in the hippocampus of FS rats treated with CBA, suggesting that CBA may inhibit neuroinflammtion in hippocampus to alleviate FS-induced brain injury.

### CBA Inhibited the Expression of BDNF and GFAP in the Hippocampus of FS Rats

To verify the anti-neuroinflammatory and anti-convulsant effect of CBA more comprehensively, the expression levels of BDNF and GFAP in rat hippocampus were investigated via immunohistochemical and qRT-PCR methods. BDNF is a protein produced in glutamatergic and glial cells, which is involved in the development of seizure (Ismail et al., 2015; Iughetti et al., 2018; Di Carlo et al., 2019). *In vivo* and *in vitro* experiments suggest that BDNF displays an epileptogenic nature by increasing neuronal excitability (Jakobsen et al., 2016; Nowroozi et al., 2021). GFAP is primarily distributed in astrocytes of the central nervous system and is a crucial marker of astrocyte activation (Chmielewska et al., 2021). The expression of GFAP increases in the process of neurological disorders related to seizure generation (Martinian et al., 2009).

**FIGURE 7:**
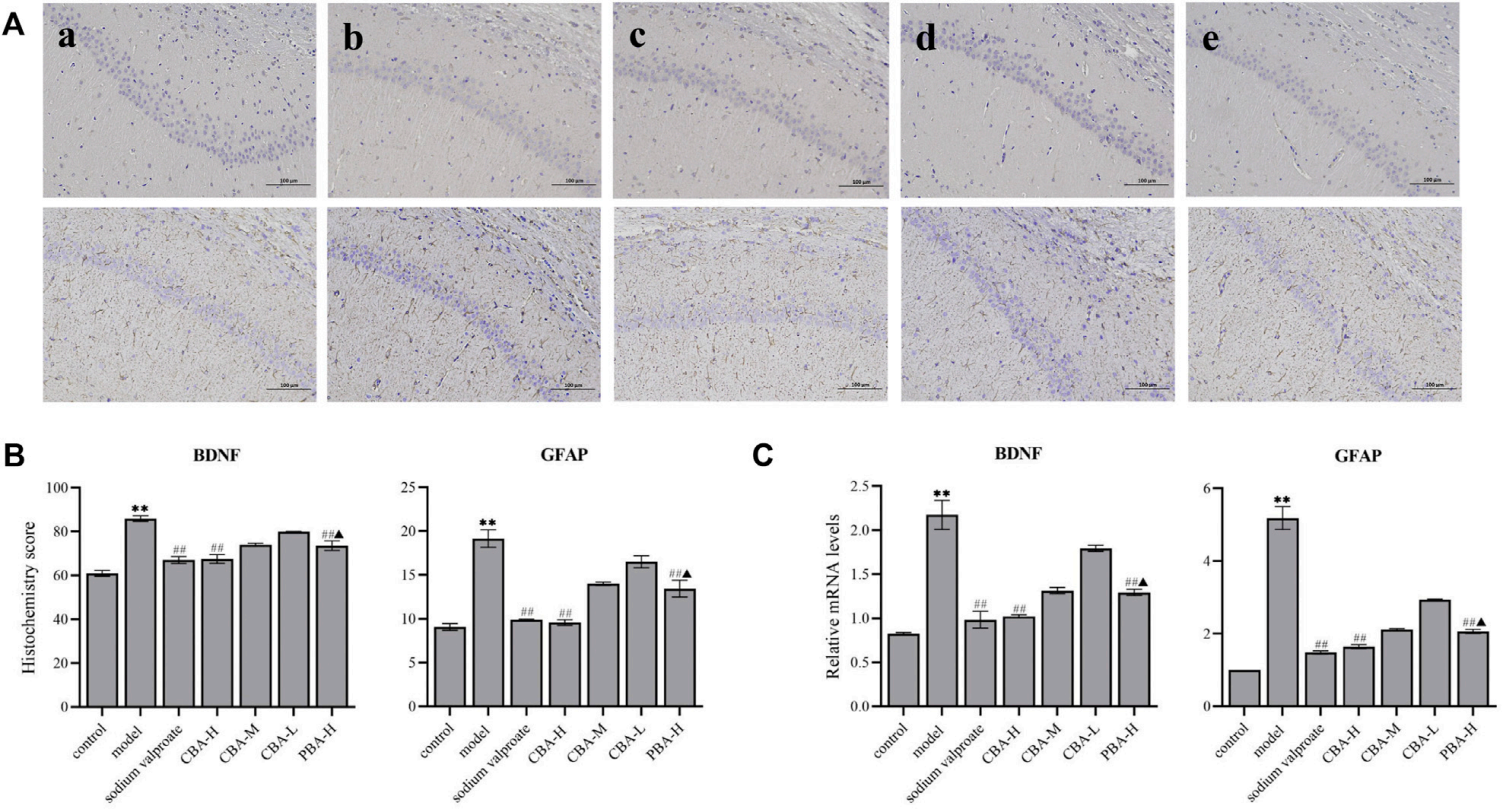
CBA inhibited the expression of BDNF and GFAP in the hippocampus of FS rats. **(A)** Immunohistochemical staining exhibiting the hippocampal expression levels of BDNF and GFAP. Original magnification ×200: (a) control; (b) model; (c) FS + sodium valproate; (d) FS + CBA-H (2.8 g/kg); and (e) FS + PBA (2.8 g/kg) groups. **(B)** The semi-quantitative analysis of the protein expression of BDNF and GFAP in the hippocampus. **(C)** The mRNA expression level of BDNF and GFAP were analyzed via qRT-PCR. Data of the histochemistry score and mRNA expressions were exhibited as the mean ± SEM. **p < 0.01 compared to that of the control group; ^##^ *p* < 0.01 compared to that of the model group; ^▲^ *p* < 0.05 compared to that of the CBA-H group.

In the present study, through the experimental data that could we find, BDNF and GFAP mRNA and protein expression levels increased observably in FS rat hippocampus (Figure 7, *p* < 0.01). While a high dose of CBA resulted in the obvious down-regulation of BDNF and GFAP levels (*p* < 0.01), which was better than that of PBA (*p* < 0.05).

The analysis of the neurotransmitters in the hippocampus suggested that the level of GABA in FS rats was lower than that of control rats, but the level of Glu was higher. The over-expression of BDNF in FS rat hippocampus verified that BDNF had pro-excitatory and anti-inhibitory effects on neurotransmitters, which was consistent with the literature report (Jakobsen et al., 2016). However, we found that CBA pre-treatment decreased the expression levels of BDNF and Glu, and increased the level of GABA. Therefore, these data demonstrate that CBA may regulate the balance of neurotransmitters by suppressing the expression of BDNF in FS rat hippocampus.

The expression level of GFAP was higher in FS rat hippocampus than in control rats, which meant that FS induced the activation of astrocytes. These activating astrocytes promoted to releasing a series of pro-inflammatory cytokines to exert an inflammatory response in the hippocampus, which was similar to previous studies (Cartmell et al., 1999). In contrast, CBA pre-treatment reversed the elevation of the expression levels of GFAP and pro-inflammatory factors in FS rat hippocampus. Hence, CBA may inhibit neuroinflammation through down-regulating the level of GFAP, and further ameliorate brain injuries induced by FS.

## Conclusion

**FIGURE 8:**
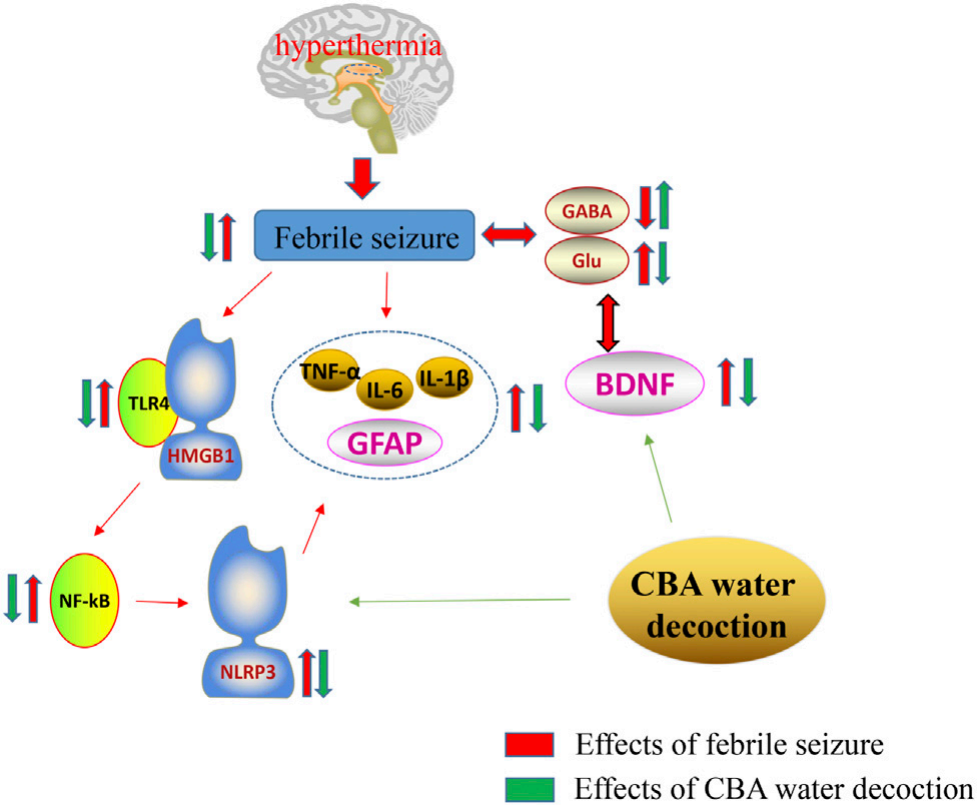
Mechanism of the neuroprotective effect of cattle bile *Arisaema* (CBA) in the febrile seizure (FS) rat model. BDNF, Brain-derived neurotrophic factor; GABA, γ-aminobutyric acid; GFAP, Glial fibrillary acidic protein; Glu, Glutamic acid; HMGB-1, Human high-mobility group box-1; IL-1β, Interleukin-1β; IL-6, Interleukin-6; NF-κB, Nuclear transcription factor-kappa B; NLRP3, Nucleotide-binding oligomerization domain- (Nol-) like receptor family pyrin domain containing 3; TLR4, Toll-like receptor 4; TNF-α, Tumor necrosis factor α.

The report of this study on the mechanism of oral administration of cattle bile *Arisaema* (CBA) aqueous extract in the treatment of febrile seizures (FS), supplements the existing literature on the ameliorative effect of bile *Arisaema* (BA) on FS. We confirm that CBA possesses better anti-convulsant effect in FS rats than that of pig bile *Arisaema* (PBA). Our data firstly demonstrate that the mechanism of CBA combating FS may involve the regulation of neurotransmitters, remission of neuronal damage in hippocampus, and suppression of neuroinflammation by inhibiting the expression of pro-inflammatory cytokines and the TLR4/NF-κB pathway (Figure 8). These achievements indicate that taking full advantage of cattle bile *Arisaema* (CBA) as a high-quality product in clinical application in the future may be better.

## Data Availability

The original contributions presented in the study are included in the article/Supplementary Material; further inquiries can be directed to the corresponding authors.

## Abbreviations

ALT, aspartate aminotransferase; AST, aspartate aminotransferase; BA, bile *Arisaema*; BCA, bicin choninic acid; BDNF, brain-derived neurotrophic factor; BUN, blood urea nitrogen; CBA, cattle bile *Arisaema*; EEG, electroencephalogram; ELISA, enzyme-linked immunosorbent assay; FS, febrile seizure; GABA, γ-aminobutyric acid; GFAP, glial fibrillary acidic protein; Glu, glutamic acid; HMGB-1, human high-mobility group box-1; IL-1β, interleukin-1β; IL-6, interleukin-6; NF-κB, nuclear transcription factor-kappa B; NLRP3, nucleotide-binding oligomerization domain (Nod)-like receptor family pyrin domain containing 3; PBA, pig bile *Arisaema*; PBS, phosphate-buffered saline; PMSF, phenylmethylsulfonyl fluoride; PVDF, polyvinylidene difluoride; qRT-PCR, quantitative real-time reverse transcription PCR; RIPA, radio-immunoprecipitation assay; SDS-PAGE, sodium dodecyl sulfate-polyacrylamide gel; TBST, tris-buffered saline Tween 20; TCM, traditional Chinese medicine; TLR4, toll-like receptor 4; TNF-α, tumor necrosis factor α; UPLC-Q-TOF-MS/MS, ultra-high-performance liquid chromatography coupled with quadrupole time-of-flight mass/mass spectrometry.
