# Supplementary figures and images for: Cattle Bile Arisaema Aqueous Extracts Protect Against Febrile Seizures in Rats Through Regulating Neurotransmitters and Suppressing Neuroinflammation
Source: Front Pharmacol. 2022 May 30;13:889055. doi: 10.3389/fphar.2022.889055 (PMC9196122; doi:10.3389/fphar.2022.889055)

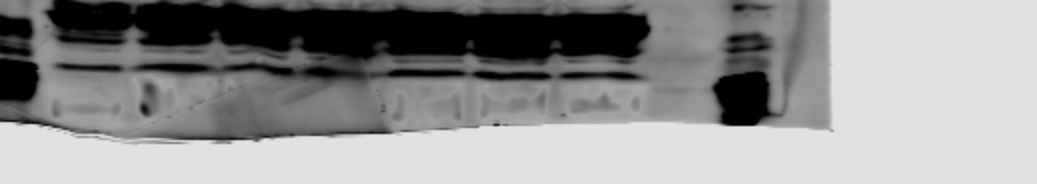

Supplement: Supplementary file 1 [file DataSheet1.ZIP › TNF-a-.tif]

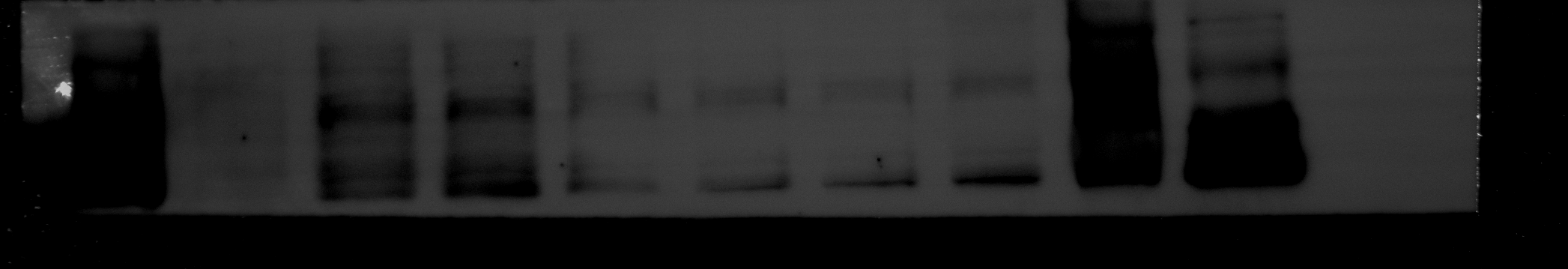

Supplement: Supplementary file 1 [file DataSheet1.ZIP › NLRP3.tif]

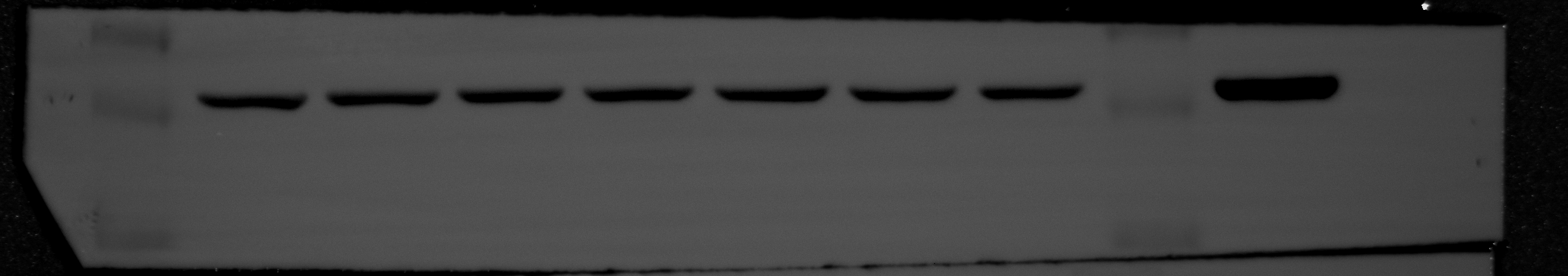

Supplement: Supplementary file 1 [file DataSheet1.ZIP › ACTIN.tif]

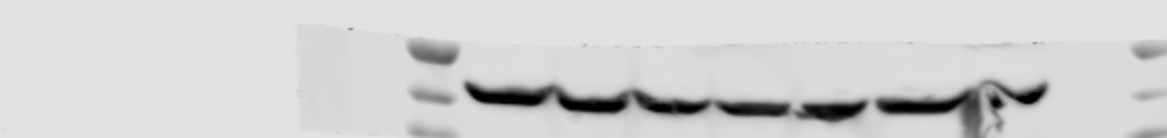

Supplement: Supplementary file 1 [file DataSheet1.ZIP › B-actin-2.tif]

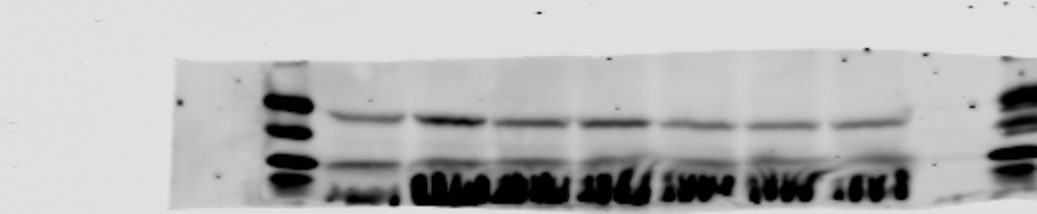

Supplement: Supplementary file 1 [file DataSheet1.ZIP › HMGB1.tif]

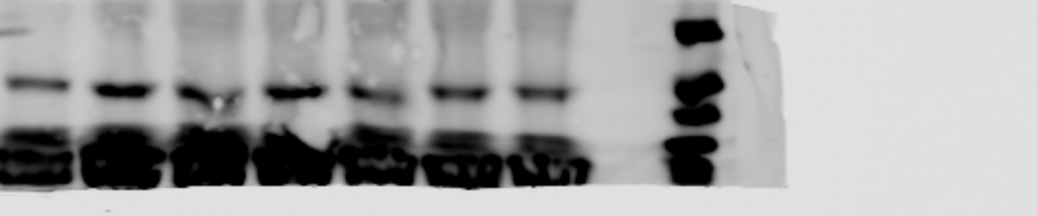

Supplement: Supplementary file 1 [file DataSheet1.ZIP › IL-1B.tif]

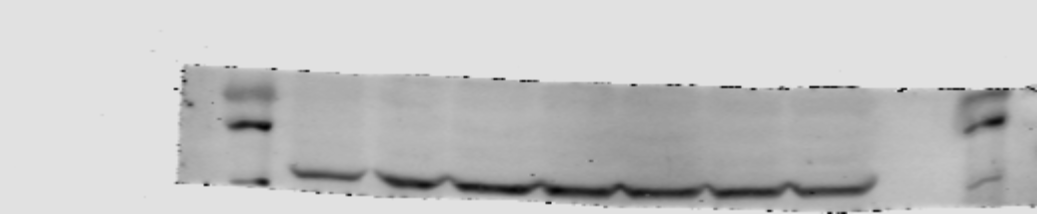

Supplement: Supplementary file 1 [file DataSheet1.ZIP › NF KB.tif]

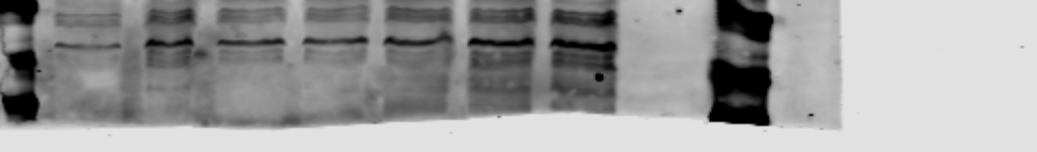

Supplement: Supplementary file 1 [file DataSheet1.ZIP › TLR4.tif]

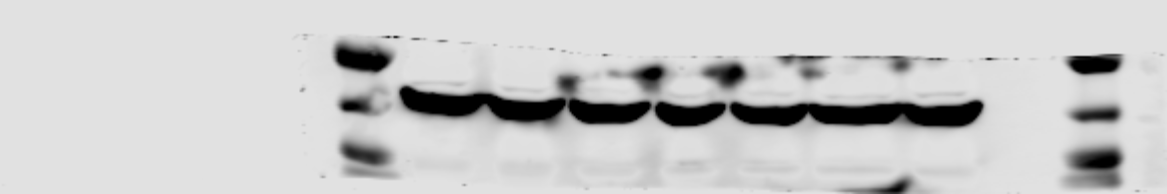

Supplement: Supplementary file 1 [file DataSheet1.ZIP › B-actin-1.tif]
